# Oxytocin Effects on Food Stimulus Processing and Food Intake in Females With or Without Binge Eating Disorder

**DOI:** 10.1002/eat.24566

**Published:** 2025-10-23

**Authors:** Julia Nannt, Mechteld M. van den Hoek Ostende, Brunna Tuschen‐Caffier, Markus Heinrichs, Daniel Sippel, Manfred Hallschmid, Jennifer Svaldi

**Affiliations:** ^1^ Department of Psychology Eberhard Karls University of Tübingen Tübingen Germany; ^2^ Department of Psychology University of Freiburg Freiburg Germany; ^3^ Department of Psychiatry and Psychotherapy University of Tübingen Tübingen Germany; ^4^ Department of Medical Psychology and Behavioral Neurobiology University of Tübingen Tübingen Germany; ^5^ German Center for Mental Health (DZPG) Tübingen Germany; ^6^ German Center for Diabetes Research (DZD) Tübingen Germany; ^7^ Institute for Diabetes Research and Metabolic Diseases of the Helmholtz Center Munich at the University of Tübingen (IDM) Tübingen Germany

**Keywords:** attentional bias, binge eating disorder (BED), food intake, hormonal contraception, menstrual cycle, oxytocin

## Abstract

**Objective:**

Binge eating disorder (BED) is maintained by increased food‐related incentive salience, which is reflected by an attentional bias for food. Oxytocin acutely attenuates this bias in patients with anorexia nervosa and reduces food intake in males with normal or increased body weight. However, results in individuals with BED have been inconclusive. We assessed the acute effect of oxytocin on food stimulus processing and reward‐driven eating behavior in females with or without BED in a double‐blind, placebo‐controlled cross‐over study.

**Method:**

Females with BED (*n* = 48) and female control participants with overweight (*n* = 46) or normal weight (*n* = 40) received intranasal oxytocin (24 IU) and, respectively, placebo, after an overnight fast and a standardized breakfast. In participants with a natural menstrual cycle, sessions were scheduled during consecutive luteal phases. Participants completed a food‐related dot‐probe task with concurrent eye tracking and a bogus taste test measuring snack intake.

**Results:**

Oxytocin compared to placebo increased dwell time bias on food stimuli in the BED relative to the overweight control group, in which this effect was reversed. Contrary to our hypothesis, oxytocin increased calorie intake across groups. Exploratory analyses indicated that the latter effect focused on females taking hormonal contraception.

**Discussion:**

These results indicate disorder‐ and, respectively, sex‐specific effects of oxytocin on food‐related incentive salience and food intake and point to a role of oxytocin in binge eating pathology. They moreover suggest that sex hormones determine the acute effect of oxytocin on eating behavior in females.

**Summary**

- Intranasal delivery of oxytocin compared to placebo increased attention to food cues in females with binge eating disorder (BED), whereas this relationship was reversed in weight‐matched females without BED.
- Surprisingly, oxytocin increased snack intake across groups; this effect focused on females taking hormonal contraception.
- These results provide support for the assumption of disorder‐ and sex‐specific effects of oxytocin on food‐related incentive salience and food intake.

## 1 Introduction

Key criteria of binge eating disorder (BED) are repeated episodes of binge eating, during which patients consume unusually large quantities of food while experiencing loss of control (American Psychiatric Association 2013). With a point prevalence rate of 1.5% in females (Erskine and Whiteford 2018), BED is the most prevalent eating disorder (Kessler et al. 2013). BED is associated with overweight and obesity (Razzoli et al. 2017); among individuals seeking obesity treatment, prevalence rates amount to 35%–57% (Ackard et al. 2007). BED is associated with a high risk of comorbid mental disorders, substantial medical, psychological and psychosocial sequelae (Hudson et al. 2007; Keski‐Rahkonen 2021), reduced health‐related quality of life (Ágh et al. 2015; Allen et al. 2013) and an increased mortality risk (Keski‐Rahkonen 2021; Smink et al. 2012). Despite persistent symptoms, less than half of individuals who qualify for the diagnosis receive treatment (Coffino et al. 2019). Moreover, meta‐analytic findings suggest that post‐treatment abstinence of binge eating is observed in only 53% of cases (Hilbert et al. 2019). This underlines the need for deeper insights into the (bio‐)psychological and neuroendocrine mechanisms that trigger and maintain binge eating and might therefore be a target of interventions that complement and improve upon current treatment options.

Cognitive models of eating disorders (Vitousek and Hollon 1990; Williamson et al. 2004) posit that episodes of binge eating are maintained by an attentional bias to disorder‐specific information such as food and food cues. This notion has received ample empirical evidence (Ralph‐Nearman et al. 2019; Schmitz et al. 2014, 2015; Svaldi et al. 2010; Werle, Sablottny, Ansorge, et al. 2024), particularly so for early attentional vigilance to high‐calorie food cues (for a systematic review see Stojek et al. 2018). Eye tracking (ET) studies have consistently demonstrated a detection bias for food stimuli (Sperling et al. 2017) and increased attentional maintenance for food stimuli in individuals with BED (Schag et al. 2013; Schmidt et al. 2016; Sperling et al. 2017). Moreover, ecological momentary assessment data obtained in females with BED showed that greater current attentional bias predicts subsequent binge eating episodes (Smith et al. 2020). A likely source of such a bias is dysfunctional processing of rewards. Incentive‐sensitization theory (Berridge 2009; Franken 2003) postulates that frequent coupling of a stimulus with a rewarding outcome leads to cue sensitization in the reward system via dopamine release, which consequently further increases the appeal of these stimuli. Food sensitization in BED can therefore be assumed to be a corollary of frequent binge eating episodes; on a behavioral level, it becomes manifest in increased attention to food stimuli and, on an emotional level, in craving and loss of control over eating (Joyner et al. 2015; Schmitz et al. 2014). Because of their relevance for the maintenance of the disorder, targeting food‐related attentional bias and, moreover, aberrant reward processing as their likely driving force may be an effective means to ameliorate binge eating behavior. In this regard, there is good reason to assume that the hypothalamic neuropeptide oxytocin might be an effective intervention (Lee et al. 2014; Nawijn et al. 2016).

Oxytocin is released by the posterior pituitary gland and controls physiological functions related to reproduction and mother‐infant interaction as well as affiliative behavior (Meyer‐Lindenberg et al. 2011) including trust, attachment, and sexual behavior (Burri et al. 2008; Domes et al. 2007; Kosfeld et al. 2005). Notably, oxytocin has turned out to be a relevant regulator of metabolic function and eating behavior (McCormack et al. 2020; Spetter and Hallschmid 2017). In rodents, oxytocin administration inhibits food intake, increases energy expenditure and enhances glucose homeostasis (Morton et al. 2012; Wu et al. 2012). Oxytocin also attenuates the rewarding aspects of food intake in animals (Sabatier et al. 2013) and, importantly, reduces food intake not only in normal‐weight rodents, but also in animals with diet‐induced obesity (Maejima et al. 2011; Olson et al. 1991; Zhang and Cai 2011). In men, oxytocin administration acutely inhibits (reward‐driven) food intake (Lawson et al. 2015; Ott et al. 2013), and this effect may be even enhanced in men with obesity compared to normal‐weight participants (Thienel et al. 2016). Negative findings notwithstanding (Melkonyan et al. 2020), it can be concluded that oxytocin contributes to the control of food intake. Studies in patients with eating disorders moreover indicate that the oxytocin system may be a suitable target of clinical interventions (Giel et al. 2018).

From a mechanistic perspective, the effects of oxytocin on food intake may result from its impact on food‐related cognitive processing. In a healthy sample of females and males, oxytocin attenuated attentional bias for food in a visual dot probe paradigm (Burmester et al. 2022). In patients with anorexia nervosa (AN), oxytocin reduced selective attention to food stimuli in the same task (Kim et al. 2014; Leppanen et al. 2017), but without immediate implications for food consumption (Kim et al. 2014). These results are in line with the assumption that beyond attentional biases, dietary restraint is an important factor that shapes eating behavior in anorexia nervosa. (Unsuccessful) cognitive restraint is of pathophysiological relevance also for BED (Blomquist and Grilo 2011), although regulatory dietary restraint is an exclusion criterion in its diagnosis. Studies on oxytocin effects in individuals with eating disorders, especially with BED are however sparse and, at the moment, inconclusive. In a female pilot sample with binge eating behavior (primarily with bulimia nervosa), which included only five females with diagnosed BED, oxytocin had no effect on the attentional processing of food stimuli, food intake or brain perfusion abnormalities (Leslie et al. 2019, 2020; Martins et al. 2020). Taken together, evidence for the impact of oxytocin on food‐related attentional processes is mixed. In addition, their mediational role in regard to the effect of oxytocin on food intake has not been tested yet.

We investigated whether oxytocin acutely reduces the attentional bias toward food cues and inhibits hedonic, that is, primarily reward‐driven food intake in females with BED. In addition, we systematically tested the assumption that oxytocin effects on food intake in BED are mediated by changes in attentional processes. In a double‐blind, placebo‐controlled, cross‐over and balanced within‐subjects comparison, females with BED and control groups of females with overweight or obesity (OWC) or normal weight without lifetime eating disorder (NWC) received intranasal oxytocin (24 IU) or placebo. Attentional bias was measured by means of a pictorial dot‐probe paradigm with concurrent eye tracking (ET). Hedonic food intake was assessed in a bogus taste test.

We hypothesized that [1] individuals with BED relative to controls display attentional bias toward food; [2] oxytocin significantly reduces this bias, and that this effect is most pronounced in the BED group; [3] oxytocin significantly reduces hedonic food intake, with this effect again being most pronounced in the BED group; [4] the oxytocin‐induced reduction of attentional bias toward food mediates the reduction in food intake. Considering the influence of the menstrual cycle on food intake (Buffenstein et al. 1995) and evidence that female sex hormones modulate the respective effect of oxytocin (Aulinas et al. 2019; Liu et al. 2020), we also investigated in exploratory analyses whether the effect of oxytocin on hedonic food intake differs between naturally cycling females and those with hormonal contraception.

## 2 Methods

### 2.1 Participants

We expected medium to large effect sizes of the presumed oxytocin effect on measures of attention (Buchheim et al. 2009; Domes et al. 2007; Guastella et al. 2008; Kim et al. 2014; Kosfeld et al. 2005; Rimmele et al. 2009) and medium effect sizes in terms of food intake (Ott et al. 2013). Based on a power of (1‐β) = 0.95, a medium effect size of *f* = 0.25, α = 0.05, and *r* = 0.30, we required 30 participants per group for the detection of within‐between interactions. To account for dropouts, we aimed to recruit 42 participants per group, totaling 126 participants.

General inclusion criteria were age between 18 and 69 years, female sex and good or corrected eyesight. Participants of the BED group needed to fulfill the diagnostic criteria for current BED according to the *Diagnostic and Statistical Manual of Mental Disorders* (American Psychiatric Association 2013). BMI criteria were ≥ 25 kg/m^2^ for the OWC group and 17.5 ≤ BMI < 25 kg/m^2^ for the NWC group. Exclusion criterion for both control groups was the presence of a current or lifetime eating disorder. Exclusion criteria that pertained to the administration of oxytocin included cardiovascular, pulmonary, or endocrine diseases including hypertension (see Supporting Information: Supplementary Methods for further exclusion criteria). Eating disorders were assessed with the Eating Disorder Examination (EDE; Hilbert and Tuschen‐Caffier 2006). Comorbidity was diagnosed with the Structured Clinical Interview for the Diagnostic and Statistical Manual of Mental Disorders I (SCID‐I and SCID‐II, 4th edition; Wittchen et al. 1997).

Participants were recruited from the community via university e‐mail newsletters, flyers and in local pharmacies and doctors' offices and received monetary compensation. All participants provided written informed consent. The authors assert that all procedures in this study comply with the ethical standards of the relevant national and institutional committees on human experimentation and with the Helsinki Declaration of 1975, as revised in 2008. All procedures were approved by institutional review board of the local university (309/2015BO1).

### 2.2 Procedure

**FIGURE 1:**
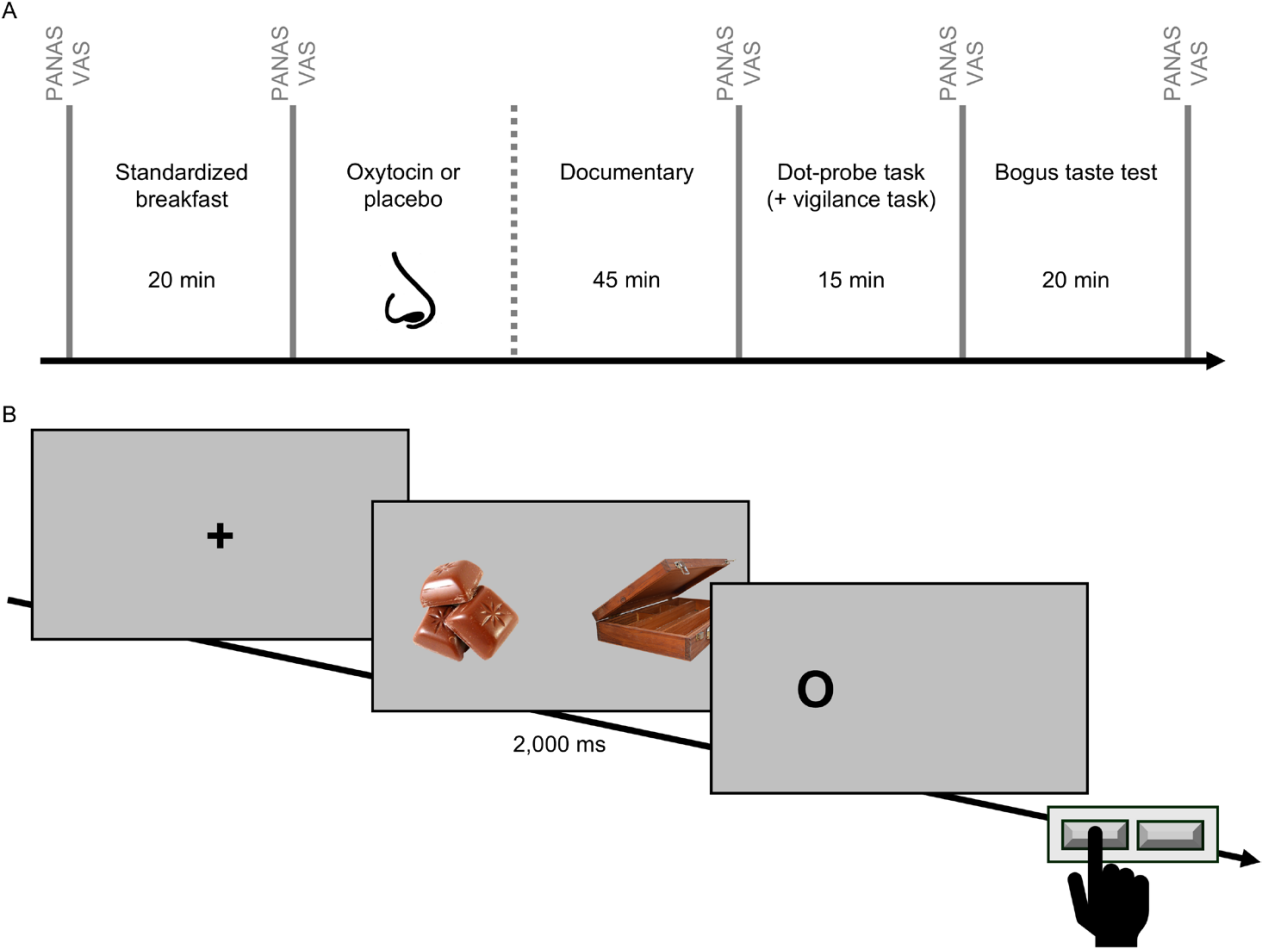
Experimental procedure (A) and dot‐probe task (B). (A) PANAS = Positive and Negative Affect Scales (Watson et al. 1988); VAS = visual analogue scale of hunger. (B) Illustrative example of a critical trial (food and neutral stimulus) of the dot‐probe task as described in the main text. The pictures presented here are for illustrative purposes only and were not used in the study.

Participants took part in two experimental sessions (oxytocin and placebo). Participants with a natural menstrual cycle attended the experimental sessions during two consecutive luteal phases; sessions were scheduled two to seven days after a positive ovulation test result (Figure S2). In post‐menopausal participants and in participants taking hormonal contraception, sessions were spaced apart approximately four weeks (Figure S2). All experimental sessions took place at 08:00 am after an overnight fast of at least 10 h. Participants received a standardized breakfast and were administered twelve intranasal puffs of oxytocin (totaling 24 IU in 0.6 mL; Ott et al. 2013) and, respectively, placebo in a double‐blind random order (see Figure 1A). To allow oxytocin to take effect, participants spent 45 min post‐administration watching animal documentaries, and afterwards completed the dot‐probe paradigm with simultaneous ET. A 5‐min Psychomotor Vigilance Task (Thompson et al. 2022) was used to assess general vigilance. Finally, participants took part in the bogus taste test. After session two, participants were reimbursed and debriefed about the purposes of the study.

### 2.3 Material

#### 2.3.1 Dot‐Probe Task

The dot‐probe paradigm assesses stimulus‐specific attention by displaying task‐irrelevant information (food or neutral stimuli) prior to depicting a task‐relevant probe (a small black circle), which is spatially aligned with one of the two previously shown stimuli, and measuring reaction times of the spatial identification of the probe (MacLeod et al. 1986). To increase reliability, we combined this task with ET (CE‐approved 250 Hz Remote Eyetracker, SensoMotoric Instruments). The main task consisted of 100 critical and 50 filler trials (randomized). In the critical trials, participants saw a high‐calorie food picture (e.g., of chocolate) presented along with a neutral picture (e.g., of a suitcase) for a total of 2000 ms, with jittered inter‐trial intervals (Figure 1B). Stimuli were selected based on two of our previous studies (Werle, Sablottny, Ansorge, et al. 2024; Werle, Sablottny, Tuschen‐Caffier, and Svaldi 2024) and were comparable in color, brightness, size, and complexity. We used a total of 20 pairs, each of which was used five times. In the filler trials 10 pairs of stationery were presented five times each. When the probe appeared on the screen after the presentation of the food/neutral stimulus pairs, participants had to indicate by key press whether it was located on the right or the left side.

Fixations were defined as periods without blinks or saccades with a duration of ≥ 100 ms. We computed total dwell time (based on fixations) per trial spent looking at food and non‐food stimuli with BeGaze 3.7 (SensoMotoric Instruments). This ET variable was selected based on other ET studies using the dot probe paradigm (Castellanos et al. 2009; Werle, Sablottny, Tuschen‐Caffier, and Svaldi 2024; Werthmann et al. 2016) and has higher reliability than reaction times (van Ens et al. 2019). To calculate attentional bias toward food stimuli, we subtracted total dwell time on neutral stimuli from total dwell time on food stimuli. Consequently, positive values reflect attentional bias toward food (see Supporting Information: Supplementary Methods and Supplementary Results for further details and ET variables).

#### 2.3.2 Bogus Taste Test

The bogus taste test took 20 min, followed an established procedure (Vöhringer et al. 2023) and comprised six high‐calorie snacks. Participants were instructed to rate the snacks on several dimensions and informed that they could eat as many snacks as they liked throughout the task. Each bowl of snacks was covertly weighed before and after the task to calculate the total amount eaten (see Supporting Information: Supplementary Methods for further details).

### 2.4 Questionnaires

Participants filled in the EDE Questionnaire (EDE‐Q; Hilbert and Tuschen‐Caffier 2016) and Beck's Depression Inventory‐II (BDI‐II; Hautzinger et al. 2006). Positive and negative mood were assessed with the Positive and Negative Affect Scales (PANAS; Watson et al. 1988). Hunger was rated on 0–100 Visual Analogue Scales (VAS). PANAS and VAS were used five times during each session (see Figure 1A). We did not assess race and ethnicity as it is recommended not to assess them in Germany.

#### 2.4.1 Data Availability

In total, 134 participants (*n*_BED_ = 48, *n*_OWC_ = 46, *n*_NWC_ = 40) completed at least one session (see Table 1 for sample characteristics and Figure S1 for flow of participants). ET data of at least one session were available for *n* = 122 participants (91%; referred to as ET sample; *n*_BED_ = 44, *n*_OWC_ = 41, *n*_NWC_ = 37). Other data sets were lost due to mechanical issues with the eye tracker or because of “staring” (see Supporting Information: Supplementary Methods). Bogus taste test data from both sessions were available for *n* = 128 participants (96%; referred to as bogus taste test sample; *n*_BED_ = 45, *n*_OWC_ = 44, *n*_NWC_ = 39). For the mediation analysis, we used the overlap between valid ET and bogus taste test data, *n* = 116 (87%). Exploratory analyses of the potential role of hormonal contraception did not include post‐menopausal females due to their very low number (reduced ET sample: *n* = 108, reduced bogus taste test sample: *n* = 113).

**TABLE 1:** Group characteristics (*n* = 134).

	BED *M* (SD)	OWC *M* (SD)	NWC *M* (SD)	Group comparison
***n***	**48**	**46**	**40**	
Age	27.78 (9.12)	33.53 (11.72)	30.73 (10.88)	*F*(2,131) = 3.34, *p* = 0.038
BMI	31.34 (7.87)	32.20 (6.05)	21.69 (1.70)	*F*(2,131) = 40.22, *p* < 0.001
BDI‐II	16.23 (11.57)	5.19 (6.47)	2.35 (2.93)	*F*(2,131) = 37.53, *p* < 0.001
EDE‐Q	2.32 (1.22)	0.81 (0.78)	0.26 (0.42)	*F*(2,131) = 64.64, *p* < 0.001

*Note*: The bold values represent the number of participants per group. For details, see the results section and figure legends (Figure 2 for eye tracking, Figure 3A for food intake, Figure 3B for food intake in dependence on hormonal contraception).

Abbreviations: BDI‐II, Beck's Depression Inventory‐II; BED, binge eating disorder; BMI, body mass index; EDE‐Q, eating disorder examination questionnaire; NWC, normal‐weight control; OWC, overweight control.

### 2.5 Statistical Analysis

Group differences in attentional bias in the placebo condition were determined with linear models (LMs). To determine a group effect, we tested a null model against the model with the main effect *Group*. To determine oxytocin effects on attentional bias and food intake, we performed linear mixed models (LMMs) with random intercepts for participants in R 4.41 (R Core Team). With a likelihood‐ratio test (LRT), we tested the predictive value of *Condition* (oxytocin, placebo) on outcome variables, and used LRT to test for the two‐fold interaction between *Condition* and *Group*. In order to test our mediation hypothesis, we conducted a mediation analysis and tested the (in)direct effect of oxytocin on food intake with the Aroian version of the Sobel test. In an exploratory analysis, we determined the interactive effect on food intake of *Condition* and *Hormonal Contraception* (naturally cycling vs. hormonal contraception) through LMMs with random intercepts for participants. Again, LRTs were used to test predictive values of the independent variables. All relevant analyses were also run with positive and negative mood according to PANAS, with eating disorder symptomatology assessed by EDE‐Q scores, with frequency of binge eating episodes in BED and, respectively, with depressive symptomatology according to BDI‐II scores included as covariates in separate analyses.

## 3 Results

### 3.1 Sample Characteristics

Participants' age ranged from 19 to 61 years (*M* = 30.6, SD = 10.8). Their BMI ranged from 19.5 to 54.6 kg/m^2^ in the BED group, from 25.1 to 48.5 kg/m^2^ in the OWC group, and from 18.1 to 24.9 kg/m^2^ in the NWC group. See Table 1 for all characteristics and respective group comparisons and Table S2 for results of mood and hunger ratings.

### 3.2 Attentional Processing

**FIGURE 2:**
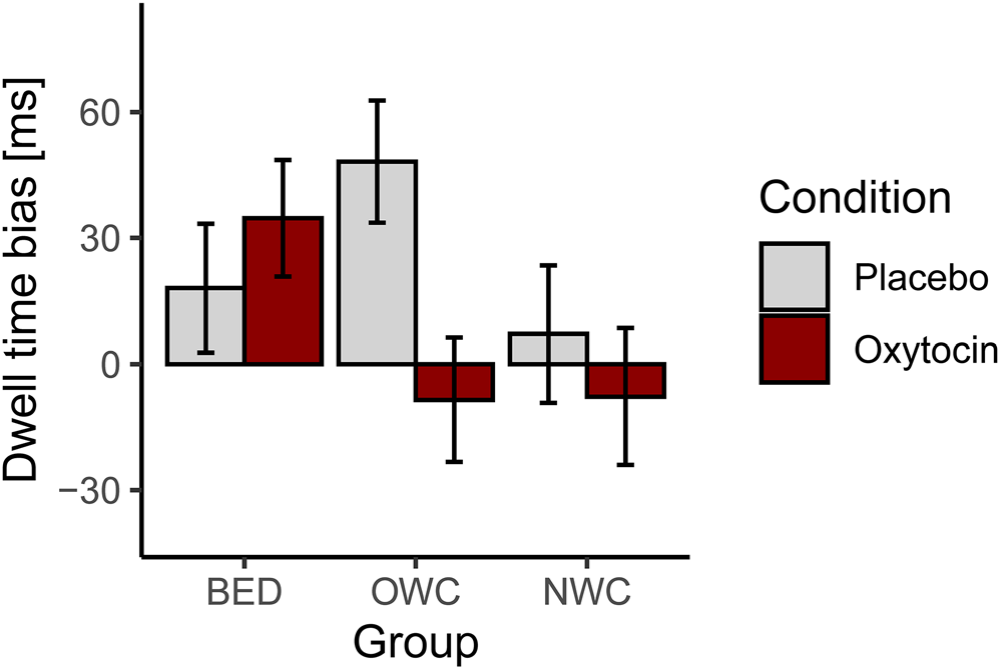
Attentional bias toward food according to group and condition. Depicted are means and standard errors. Group × Condition: *χ* ^2^[2] = 6.24, *p* = 0.044 in the eye tracking sample (*n* = 122). BED = binge eating disorder, *n* = 44, OWC = overweight control, *n* = 41, NWC = normal‐weight control, *n* = 37; results of *n* = 12 individuals did not enter analyses due to mechanical issues with the eye tracker or because there were not enough fixations on the areas of interest. The placebo condition is shown in light gray and the oxytocin condition in red (dark gray).

Analyses of attentional processing of food as reflected by dwell time bias toward food stimuli in the placebo conditions did not yield group differences (*F* [2,3675] = 2.13, *p =* 0.119). In analyses of potential oxytocin effects on dwell time bias, the interaction *Group* × *Condition* significantly improved model fit in comparison to the main effect models (LRT, χ^2^[2] = 6.24, *p* = 0.044). Fixed effects analyses indicated that the effect of oxytocin vs. placebo was significantly different in the BED compared to the OWC group (*t*[7171.94] = 2.46, *p* = 0.014). Thus, relative to OWC, individuals with BED showed an increase in attention toward food upon oxytocin compared to placebo administration, with a relatively reversed pattern of an oxytocin‐induced reduction in attentional bias toward food in participants of the OWC group (Figure 2). A similar pattern in the comparison of BED and NWC groups just failed to reach significance (*p* = 0.052), whereas oxytocin did not differentially affect attentional bias in OWC vs. NWC (*p* = 0.775). See Supporting Information: Supplementary Results for further details on attentional processing and results of the vigilance task and dot probe task reaction times, two additional ET variables and PANAS and VAS data.

### 3.3 Food Intake

**FIGURE 3:**
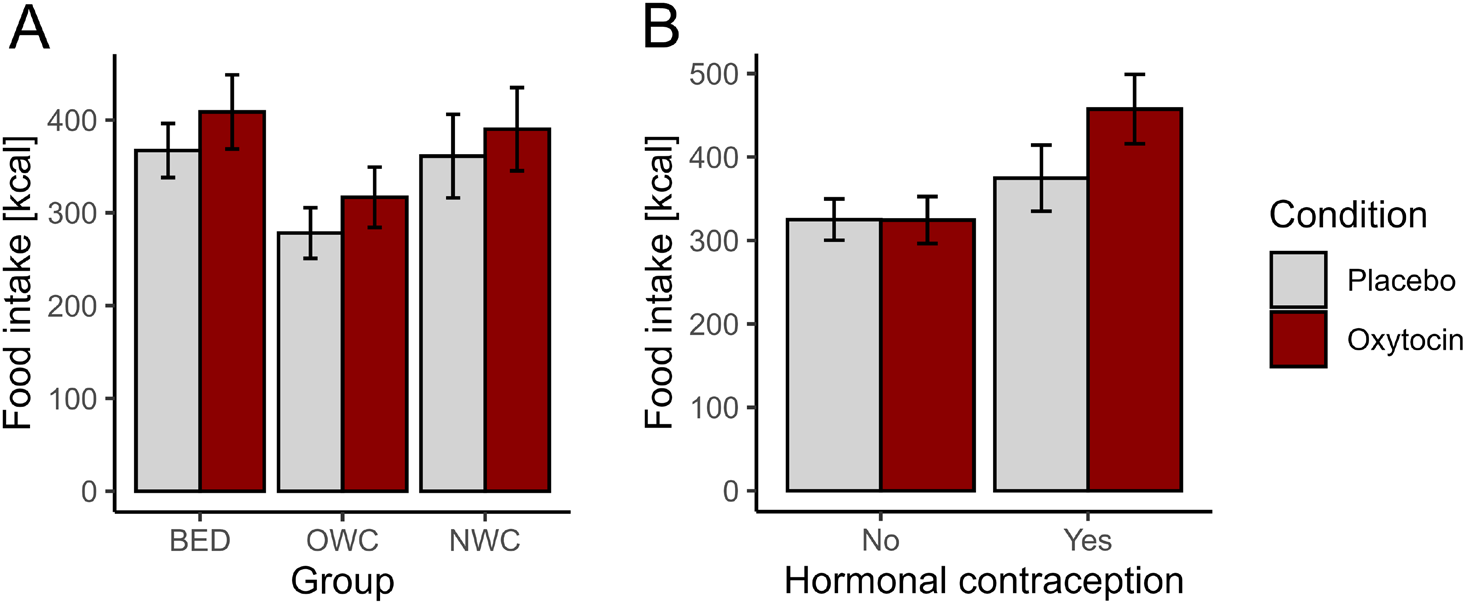
Calories consumed according to group and condition (A) and to hormonal contraception and condition (B). Depicted are means and standard errors. (A) *Condition*: LRT, χ^2^[1] = 4.25, *p* = 0.039 in the bogus taste test sample (*n* = 128). BED = binge eating disorder, *n* = 45, OWC = overweight control, *n* = 44, NWC = normal weight control, *n* = 39. The bogus taste test sample did not include participants who attended only one of the two sessions (*n* = 6). (B) *Hormonal Contraception* × *Condition*: χ^2^[1] = 4.72, *p* = 0.030 in the reduced bogus taste test sample (*n =* 113). No hormonal contraception: *n =* 72, hormonal contraception: *n* = 41. Post‐menopausal females (*n* = 15) were not included. The placebo condition is shown in light gray and the oxytocin condition in red (dark gray).

Contrary to our expectation, oxytocin compared to placebo increased food intake, as reflected by a significant main effect of *Condition* (LRT, χ^2^[1] = 4.25, *p* = 0.039; Figure 3A). Against our hypothesis of a preferential oxytocin effect on food intake in individuals with BED, the interaction *Group* × *Condition* did not improve model fit (*p* = 0.957), nor did *Group* have a significant impact as a main factor (*p* = 0.104). Mediation analyses did not yield signs of a significant contribution of changes in attentional bias to changes in food intake (*p* = 0.877). Our exploratory analysis indicated a main effect of *Hormonal Contraception* on food intake (LRT, χ^2^[1] = 4.60, *p* = 0.032), that is, higher food intake in females on hormonal contraception compared to naturally cycling females. In these exploratory analyses, oxytocin compared to placebo significantly increased food intake only in females taking hormonal contraception, whereas calorie intake was not affected by oxytocin in naturally cycling females (LRT, χ^2^[1] = 4.72, *p* = 0.030 for *Hormonal Contraception* × *Condition*; Figure 3B). The addition of *Group* did not improve model fit (*p* = 0.173).

In all relevant analyses, the pattern of significant results was not essentially altered by including PANAS mood ratings, EDE‐Q scores, frequency of binge eating episodes, or BDI‐II scores as covariates in the models.

## 4 Discussion

We investigated the acute effect of oxytocin on attentional bias and hedonic food intake in females with BED compared with female controls with overweight or normal weight. Contrary to our hypothesis, oxytocin increased rather than reduced attention to food vs. neutral cues in individuals with BED relative to OWC, who in comparison displayed the expected oxytocin‐induced decrease. We did not observe the assumed general group differences in attention bias. In striking contrast to our expectation and to previous findings in males (Lawson et al. 2015; Ott et al. 2013; Thienel et al. 2016), oxytocin increased rather than decreased food intake, and did so across groups. Exploratory analyses indicated that this effect, which was not mediated by altered attentional processing, focused on females taking hormonal contraception.

Contrary to prior evidence for a food bias in patients with BED (Schmitz et al. 2014; Svaldi et al. 2010), but consistent with another study (Leslie et al. 2020), we found comparable rather than differential food‐related attentional processing in individuals with BED, OWC and NWC. In comparison to the reference samples cited above, our patients tended to be younger and to display less overweight and a relatively milder eating disorder pathology. This was most likely due to the exclusion criteria imposed by the use of oxytocin, which led to the exclusion of 74 potential participants because of hypertension, which is associated with older age and higher BMI (Speer et al. 2021) and is often comorbid with BED (Keski‐Rahkonen 2021). That potential participants with longer disease duration and, possibly, more severe symptomatology were excluded is also reflected in the relatively low mean EDE‐Q score of our sample (Hilbert et al. 2007).

Oxytocin did not attenuate food bias in BED. Instead, individuals with BED relative to those of the OWC group spent more time looking at food after oxytocin vs. placebo administration. This interaction effect appears to be food‐specific because oxytocin generally improved vigilance (see Supporting Information: Supplementary Results). To date, only one pilot study has examined the influence of oxytocin on food bias in a combined sample of patients with BED or bulimia nervosa and NWC, showing increased attention toward food after oxytocin vs. placebo across groups (Leslie et al. 2020). These seemingly paradoxical effects in females with BED may be explained by an increase in reward sensitivity via dopaminergic pathways following oxytocin administration (Spetter et al. 2018) leading to cue sensitization (Schmitz et al. 2014) and, eventually, increased attention to food. In the OWC group, in contrast, oxytocin attenuated food bias relative to the BED group. Individuals with overweight but without BED have better inhibitory capacities and lower food‐related impulsivity than individuals without BED (Hege et al. 2014; Svaldi et al. 2014). Oxytocin reduces food‐related bias also in patients with AN (Kim et al. 2014), who have high inhibitory capacities (Weinbach et al. 2020) and can therefore be considered the opposite of people with BED on the spectrum of (loss of) control over eating. Likewise, oxytocin‐induced increases in the activity of brain regions that mediate cognitive control have been found in healthy male subjects with normal weight (Spetter et al. 2018) and males with overweight or obesity (Plessow et al. 2018). Thus, differences in the balance between reward‐processing and cognitive control pathways may explain why oxytocin‐induced increases in reward sensitivity lead to increased attention to food in BED, but not in individuals without BED, who might benefit to a greater extent from concurrent oxytocin‐triggered improvements in inhibitory skills.

Contrary to what we hypothesized, oxytocin increased food intake across all groups, while previous studies have rather shown a reduction (or no change) in food intake after intranasal oxytocin delivery (Lawson et al. 2015; Ott et al. 2013; Spetter et al. 2018; Thienel et al. 2016). Notably, almost all those studies were conducted in males. In females, one proof‐of‐concept study showed no effect of oxytocin (Leslie et al. 2019). A second study found that females under stress ate fewer sweet snacks, but the same number of salty snacks after oxytocin compared to placebo administration (Burmester et al. 2019). Experiments in female rats indicated that a single injection of oxytocin results in an increase in food intake (Björkstrand and Uvnäs‐Moberg 1996). However, a chronic systemic infusion of oxytocin has been observed to reduce food intake in female mice and rats (Dodson et al. 2024; Maejima et al. 2017).

The conclusion that oxytocin's acute effect on food intake depends on biological sex and, possibly, on hormonal factors, is supported by related reports. First, in neuroimaging studies oxytocin reduced functional connectivity in areas involved in motivational and affective processes in males, whereas females displayed oxytocin‐induced increases (Bethlehem et al. 2017; Kerem and Lawson 2021). In terms of food intake, this pattern may explain why oxytocin increases the reward value of food and, consequentially, calorie consumption in females but not males. This assumption is consistent with general sex differences in the physiology of eating and in cognitive control processes that act on homeostatic and hedonic drivers of food intake, which may arise from differential effects of estrogens compared to androgens (Sample and Davidson 2018). Second, hormonal contraceptives increase plasma levels of oxytocin (Wikström Frisén et al. 2017), with possible implications for subjective hunger (Aulinas et al. 2019).

Considering oxytocin's possible interaction with sex hormones in the regulation of food intake, we conducted exploratory post hoc analyses to examine whether hormonal contraception modulated the influence of oxytocin on food intake. These exploratory analyses indicated that oxytocin increased food intake specifically in females taking hormonal contraception, who in general ate more than naturally cycling females, which excludes a ceiling effect. Although eating behavior is susceptible to hormonal changes (Buffenstein et al. 1995), the potential modulatory influence of the menstrual cycle on food‐related oxytocin effects and contraceptive use has not been systematically addressed. The absence of oxytocin effects on food intake in females in the luteal phase of their cycle—as well as the surprising oxytocin‐induced increase in food intake during hormonal contraception—provide robust new evidence for the idea that oxytocin's regulatory role in eating behavior may be sex‐specific and depend on the menstrual cycle (Aulinas et al. 2019; Kerem and Lawson 2021; Liu et al. 2020). Notably, the administration of exogenous estrogen to rats inhibited the anorexigenic function of oxytocin, but did not reverse it (Liu et al. 2020). Given the current lack of mechanistic insights, further studies are needed to determine how biological sex, the menstrual cycle and hormonal contraception modulate the metabolic action profile of oxytocin.

Beyond lack of a pre‐registration, there are several other limitations to the interpretation of our results. First, as outlined above, we had to exclude individuals with clinically manifest hypertension because of the oxytocin intervention, which might have affected the generalizability of our results, in particular with regard to the BED sample. Moreover, we were not able to examine whether BMI may have influenced the findings within the BED group (i.e., normal‐weight vs. overweight) because of the highly unequal subgroup sizes. Furthermore, our analyses were not statistically controlled for BMI because ANCOVA is not designed to control for naturally occurring group differences (Jamieson 2004; Miller and Chapman 2001) as found between individuals with BED compared to individuals without BED (Ivezaj et al. 2014; Telch et al. 1988). Future studies should examine the potential interplay between BED diagnosis and BMI more systematically, for instance by recruiting larger samples of normal‐weight participants with BED and individuals with overweight and BED.

Second, as only females participated in the current study, we cannot directly compare the effect of oxytocin on hedonic food intake between males and females. However, oxytocin dosing and administration, and the implementation of the bogus taste test were modeled on previous studies in male participants (Ott et al. 2013; Spetter et al. 2018; Thienel et al. 2016), which suggests that oxytocin has differential effects on eating behavior in males and females. Still, future studies are needed that test females and males in the same study.

Third, all females without hormonal contraceptives were studied in the luteal phase. Therefore, no conclusions can be drawn about the influence of different phases of the cycle. Since the progesterone level possibly interacts with the effect of exogenous oxytocin, follow‐up studies should look at females in the follicular phase (Buffenstein et al. 1995). In addition, the relevance of the type of hormones used for hormonal cycle control will need to be explored.

While oxytocin administration has been discussed and investigated as a promising intervention in individuals with BED and overweight or obesity (Niu et al. 2021; Plessow et al. 2024), our findings shed new light on this approach because they suggest that oxytocin's role in food intake control is even more complex than so far assumed and that its delivery may come with unintended effects. Therefore, a better understanding of its interactions with biological sex and sex hormones is necessary and will inform the development of therapeutic administration options in (female) populations. In this regard, our results offer new insights into oxytocin's effect both on the attentional processing of food and on food intake itself. Contrary to our hypotheses, oxytocin increased calorie intake in females taking hormonal contraceptives and intensified rather than attenuated bias toward food in females with BED relative to OWC, in which it triggered the reverse effect. These unexpected findings might stem from an enhancing effect of oxytocin on incentive salience and, in terms of attentional processes, from a disorder‐specific interplay of incentive salience with inhibitory resources. Testing these hypotheses should be a primary target of future studies into oxytocin's role in disordered eating. Furthermore, as the impact of oxytocin on food intake seems to depend on biological sex and sex hormones, (biological) sex should no longer be neglected in research into oxytocin's metabolic action profile and, particularly, in clinical trials that assess the efficacy of oxytocin administration in real‐world settings. In fact, the mixed evidence for beneficial metabolic effects of long‐term oxytocin delivery found in mixed‐sex samples (Plessow et al. 2024; Zhang et al. 2013) calls for the consideration of potential intervening factors including sex hormones and sex.

## Author Contributions

**Julia Nannt:** data curation, formal analysis, investigation, project administration, visualization, writing – original draft. **Mechteld M. van den Hoek Ostende:** formal analysis, visualization, writing – original draft. **Brunna Tuschen‐Caffier:** funding acquisition, validation, writing – review and editing. **Markus Heinrichs:** funding acquisition, validation, writing – review and editing. **Daniel Sippel:** investigation. **Manfred Hallschmid:** conceptualization, funding acquisition, writing – original draft, methodology, supervision, validation. **Jennifer Svaldi:** conceptualization, funding acquisition, writing – original draft, methodology, validation, resources, supervision.

## Conflicts of Interest

The authors declare no conflicts of interest.

## Supporting information


**Data S1:** eat24566‐sup‐0001‐supinfo.docx.

## Data Availability

The data that support the findings of this study are openly available in OSF at https://osf.io/e4vqx/.
