# Supplementary material for: Oxytocin Effects on Food Stimulus Processing and Food Intake in Females With or Without Binge Eating Disorder
Source: Int J Eat Disord. 2025 Oct 23;59(2):249–59. doi: 10.1002/eat.24566 (PMC12884240; doi:10.1002/eat.24566)
Supplement: Supplementary file 1 — Data S1: eat24566‐sup‐0001‐supinfo.docx. [file EAT-59-249-s001.docx]

**Supplement**

Oxytocin effects on food stimulus processing and food intake in females with or without binge eating disorder

**Table of contents**

[**Supplementary methods** 2](#_Toc204851784)

[**Participants** 2](#_Toc204851785)

[**Material** 2](#_Toc204851786)

[*Dot-probe task* 2](#_Toc204851787)

[*Bogus taste test* 3](#_Toc204851788)

[*Psychomotor Vigilance Task (PVT)* 3](#_Toc204851789)

[*Questionnaires* 4](#_Toc204851790)

[**Data availability and analysis** 4](#_Toc204851791)

[**Additional analyses** 4](#_Toc204851792)

[**Supplementary results** 6](#_Toc204851793)

[**Early attentional processing after placebo administration** 6](#_Toc204851794)

[**Effect of oxytocin on early attentional processes** 6](#_Toc204851795)

[**Mediation analysis** 6](#_Toc204851796)

[**Reaction times in the dot-probe and the vigilance tasks** 6](#_Toc204851797)

[**Reaction times in the vigilance task according to hormonal contraception** 6](#_Toc204851798)

[**Location of first fixation in the dot-probe task according to hormonal contraception** 7](#_Toc204851799)

[**Supplementary Table S1.** Hormonal status according to experimental group. 8](#_Toc204851800)

[**Supplementary Table S2.** Positive and Negative Affect Scale and VAS hunger ratings. 9](#_Toc204851801)

[**Supplementary Figure S1.** Flow of participants 10](#_Toc204851802)

[**Supplementary Figure S2.** Timing of experimental sessions based on hormonal status 11](#_Toc204851803)

[**Supplementary Figure S3.** Reaction times in the vigilance task according to group and condition*.* 12](#_Toc204851804)

[**Supplementary Figure S4.** Reaction times in the vigilance task according to hormonal contraception and condition. 12](#_Toc204851805)

[**Supplementary references** 13](#_Toc204851806)

# **Supplementary methods**

# **Participants**

In all groups, exclusion criteria were the presence of bipolar disorder, substance use disorder, schizophrenia or current psychosis, severe suicidal ideation, borderline personality disorder, current pregnancy or lactation, shift work with night shifts, neurological or gastrointestinal diseases, other physical diseases (including hormonal diseases such as polycystic ovary syndrome, PCOS) or impairments that would prevent the completion of the study, smoking more than five cigarettes per day, insufficient German proficiency, current treatment for eating disorders, participation in weight reduction programs or respective experiments. Additionally, participants were excluded following initial enrollment in case they displayed markedly irregular menstrual cycles and ovulation could not be ascertained by the ovulation tests. Supplementary Table S1 gives an overview of participants according to hormonal status.

# **Material**

# *Dot-probe task*

To prevent participants from changing their natural eye movements, authorized deception specified pupil dilation as our research interest. The task was presented on a computer screen in a dimly lit room.

Each trial of the dot-probe task started with the presentation of a fixation cross in the middle of a light gray screen (RGB 192, 192, 192). To proceed with the trial, the eye tracker needed to measure a fixation of variable duration between 1500 ms and 2400 ms. After such a fixation, two stimulus pictures were presented at each side of the screen for a total of 2000 ms. The pictures were then replaced by a probe, consisting of a small black circle that spatially aligned with one of the two previously shown pictures (see Figure 1B in the main document). Inter-trial intervals were between 1500 ms and 2400 ms. The task was to indicate as quickly and accurately as possible by key press whether the probe appeared on the right or the left screen side. Once participants pressed the corresponding button, the next trial started automatically. The location of the probe was balanced between trials.

After four practice trials, the main task consisted of 150 trials, 100 of which were critical and 50 were filler trials. In the critical trials, participants saw a high-calorie food stimulus (e.g., chocolate) presented along a neutral stimulus (e.g., suitcase). Stimuli that were presented alongside each other were pre-tested to be similar in color, brightness, size, and complexity. We used a total of 20 pairs, each of which was used five times. In the filler trials ten pairs of stationery were presented five times each. Trials were pseudo-randomized, so that the same image was not presented twice in a row, and the dot probe did not appear in the same location more than four times in a row.

Filler trials were excluded from analysis. Fixations were defined as periods without blinks or saccades with a duration of ≥ 100 ms. We computed total dwell time (based on fixations) per trial spent looking at food and non-food stimuli with BeGaze 3.7 (SensoMotoric Instruments). This eye tracking (ET) variable was selected based on other ET studies using the dot probe paradigm (Castellanos et al., 2009; Werle et al., 2024; Werthmann et al., 2016). In analyses of the ET variable fixations in critical trials, fixations were only regarded as valid if at least one fixation was on either one of the stimuli. Critical trials with no such fixation were excluded from analysis (40% of all trials). Participants with fewer than 20 trials available to calculate ET measures were identified as “starer” and were excluded, as these indices would be highly biased. This led to the exclusion of ET data sets from *n* = 18 participants for session 1 and *n* = 17 participants for session 2, which is slightly more than in comparable studies that applied the same paradigm (Werthmann et al., 2016, 2019; see Supplementary Figure S1 for details).

# *Bogus taste test*

Snacks offered in the bogus taste test were mini pretzels, 401 kcal/100 g; chocolate biscuits, 479 kcal/100 g; salty coated peanuts, 547 kcal/100 g; chocolate chip cookies, 502 kcal/100 g; crisps, 508 kcal/100 g; chocolate-covered peanuts, 495 kg/100 g. We presented the snacks in large yellow bowls spaced in equidistance on a round turning table. The arrangement of the snack bowls on the table was randomized between participants, but intra-individually consistent over sessions. During the test, participants sat next to the turning table. As a cover story, participants were instructed to rate the snacks on several dimensions (e.g., taste). Furthermore, we instructed participants that they could eat ad libitum from the remaining snacks until the end of the task. Participants rated the snacks in terms of appearance, taste, intensity, and flavor characteristics on 10-cm-long visual analog scales (VAS) anchored at “not at all” and “very”. We informed participants they had 20 minutes to complete the task and that they would be left alone in the room.

# *Psychomotor Vigilance Task (PVT)*

A 5-minute PVT was used to measure vigilance-based reaction times as a control measure to detect potential oxytocin-induced changes in general vigilance. The PVT is known as the “gold standard” vigilance task (Khitrov et al., 2014) and the 5-minute version has a high test-retest reliability (Thompson et al., 2022). During the test participants were alone in the laboratory. In each trial, a placeholder (“8888” displayed in thin digits) was shown first, then, after a random interval between 2 s and 10 s, a millisecond counter started. Participants then had to press the spacebar as fast as possible. We used reaction times as outcome variable.

# *Questionnaires*

The Eating Disorder Examination Questionnaire (EDE-Q; Hilbert & Tuschen-Caffier, 2016) was used to assess eating disorder pathology, and Beck’s Depression Inventory-II (BDI-II; Hautzinger et al., 2006) to evaluate severity of depressive symptoms. Both questionnaires have good psychometric properties (Hilbert et al., 2007; Kühner et al., 2007). The Positive and Negative Affect Scale (PANAS) consists of 20 items which are answered on a five-point Likert scale. Half of the items measures positive and the other half measures negative affect. Additionally, participants answered questions on their current hunger on 0-100 Visual Analogue Scales (VAS) anchored at “not at all” and “very much” (Supplementary Table S2).

# **Data availability and analysis**

In contrast to the dot probe paradigm, which includes several trials, the bogus taste test only provides a single estimation of food intake per session. Since this would allow personal food preferences of participants with only a single session to affect mean estimates, these participants were not considered in this analysis (*n* = 6). Note that additionally excluding participants who attended only one session from the ET analyses did not robustly change our results.

Vigilance data (from the PVT-5) and dot-probe reaction time data of at least one session were available for the total sample (*N* = 134). For the exploratory analysis we again analyzed a reduced sample (reduced vigilance sample: *n* = 118). Linear mixed models (LMMs) with random intercepts for participants were performed using the lme4-package (Bates et al., 2015) in R 4.41 (R Core Team).

# **Additional analyses**

The following ET measures were analyzed in addition to dwell time bias: (1) Location of first fixation (percentage of first fixations on food), (2) duration of first fixation on food. We conducted the same statistical analyses for these ET variables as for dwell time bias.

To determine the effect of oxytocin on dot-probe and vigilance task reaction times in the total sample (*N* = 134), we performed linear mixed models with random intercepts for participants. As described in the main document, we tested a null model (LMM0) against each of the models with a single main effect (LMM1 and LMM2). In turn, we tested the models with one main effect against the model with both main effects (LMM3). Finally, we tested the model with the main effects against a model with both main effects and their interaction.

# **Supplementary results**

# **Early attentional processing after placebo administration**

We did not find differences between the BED, OWC and NWC groups in the attentional processing of food as reflected by location of first fixation (*F*[2,112] = 2.19, *p =* .116) and duration of first fixation on food (*F*[2,112] = 2.17, *p =* .118).

# **Effect of oxytocin on early attentional processes**

For the location of first fixation, we found neither a main effect of *Condition* nor an interaction of *Group* and *Condition* (*p* > .085 for all likelihood-ratio tests [LRTs]). Similarly, oxytocin vs. placebo administration did not affect the duration of first fixation on food in general and in dependence of the experimental group (all *p* > .124 for *Condition* and *Group* × *Condition*).

# **Mediation analysis**

The mediation analysis did not yield signs of significant contributions to changes in food intake of the location of first fixations, and the duration of first fixations on food (*p* > .642 for all analyses). In line with the results of the mediation analysis of dwell time bias (see main document), this outcome indicates that the influence of oxytocin on food intake was not mediated by effects on attentional processes.

# **Reaction times in the dot-probe and the vigilance tasks**

For the reaction times in the dot-probe task, LTRs did not indicate a main effect of *Condition* or *Group*, nor was there an interaction between the two (all *p*s > .086). In contrast, LRT indicated a main effect of *Condition* on reaction times in the vigilance task (χ^2^[1] = 4.81, *p* = .028), with shorter reaction times after oxytocin relative to placebo (Supplementary Figure S3). There was no main effect of *Group* nor an interaction between the two factors (all *p* > .471).

# **Reaction times in the vigilance task according to hormonal contraception**

LRT indicated an interaction effect of *Hormonal Contraception* and *Condition* on reaction times in the vigilance task (χ^2^[1] = 5.77, *p* = .016), in the absence of any main effects (all *p*s > .153). Females without hormonal contraception achieved shorter reaction times under oxytocin, while females with hormonal contraception achieved longer reaction times under oxytocin (Supplementary Figure S4).

# **Location of first fixation in the dot-probe task according to hormonal contraception**

LRT indicated a main effect of *Condition* on location of first fixation on food (χ^2^[1] = 4.50, *p* = .034), in the absence of any other effects (all *p* > .220) in the subsample without post-menopausal females. Specifically, oxytocin compared to placebo reduced the location of first fixation on food. There were no main effects or interactions of *Condition* and *Hormonal Contraception* on duration of first fixation, dwell time bias or dot-probe reaction times (all *p*s > .126).

# **Supplementary Table S1.** Hormonal status according to experimental group.

|  | Total sample  *(n* = 134) | BED | OWC | NWC |
| --- | --- | --- | --- | --- |
| Naturally cycling | 76 | 34 | 24 | 18 |
| Hormonal contraception | 42 | 12* | 14 | 16* |
| *Combined* | 33 | 7 | 13 | 13 |
| *Progestogen-only* | 7 | 4 | 1 | 2 |
| Post-menopausal | 16 | 2 | 8 | 6 |

Combined contraceptives are defined as containing synthetic estrogen and synthetic progesterone. Logistic regression indicated no significant group differences in hormonal status (*χ*²[1] = 2.69, *p* = .101). BED = Binge Eating Disorder, *n* = 48, OWC = Overweight Control, *n* = 46, NWC = Normal-Weight Control, *n* = 40. *One person in the BED and one person in the NWC group were unable to state which oral contraceptive they were taking.

# **Supplementary Table S2.** Positive and Negative Affect Scale and VAS hunger ratings.

|  |  | BED | | OWC | | NWC | |
| --- | --- | --- | --- | --- | --- | --- | --- |
|  |  | Placebo | Oxytocin | Placebo | Oxytocin | Placebo | Oxytocin |
| T1 |  |  |  |  |  |  |  |
|  | Positive Affect | 27.0 (5.86) | 26.9 (5.90) | 28.7 (6.23) | 28.6 (6.08) | 28.2 (6.05) | 29.6 (6.46) |
|  | Negative Affect | 12.7 (3.14) | 12.2 (2.92) | 11.6 (2.37) | 11.6 (4.40) | 11.1 (1.62) | 11.1 (2.19) |
|  | Hunger | 58.8 (24.8) | 57.6 (23.4) | 53.7 (28.0) | 58.7 (28.1) | 62.0 (24.5) | 65.7 (19.7) |
| T2 |  |  |  |  |  |  |  |
|  | Positive Affect | 26.7 (6.63) | 26.9 (6.97) | 29.7 (6.06) | 30.0 (6.44) | 29.3 (6.43) | 30.3 (6.59) |
|  | Negative Affect | 11.8 (2.74) | 11.8 (3.45) | 10.8 (1.48) | 10.5 (1.69) | 10.3 (0.46) | 10.4 (0.79) |
|  | Hunger | 8.18 (8.4) | 7.62 (8.5) | 7.55 (12.6) | 8.32 (11.7) | 11.1 (16.8) | 11.8 (15.6) |
| T3 |  |  |  |  |  |  |  |
|  | Positive Affect | 24.2 (6.30) | 24.0 (6.73) | 26.8 (6.99) | 27.3 (7.54) | 26.2 (6.33) | 28.4 (7.29) |
|  | Negative Affect | 10.7 (1.27) | 10.6 (0.95) | 10.4 (0.98) | 10.3 (0.54) | 10.3 (0.64) | 10.2 (0.55) |
|  | Hunger | 10.2 (12.9) | 13.1 (15.7) | 7.82 (12.0) | 8.11 (12.2) | 11.3 (12.9) | 11.2 (12.7) |
| T4 |  |  |  |  |  |  |  |
|  | Positive Affect | 24.0 (6.17) | 23.98 (6.95) | 28.5 (7.57) | 28.1 (7.89) | 26.9 (8.59) | 28.5 (8.35) |
|  | Negative Affect | 10.7 (1.30) | 11.0 (1.54) | 10.6 (1.33) | 10.5 (1.07) | 10.23 (0.48) | 10.4 (0.63) |
|  | Hunger | 13.7 (15.5) | 14.3 (18.1) | 7.41 (10.4) | 8.59 (12.6) | 12.2 (15.6) | 13.9 (16.7) |
| T5 |  |  |  |  |  |  |  |
|  | Positive Affect | 25.2 (6.24) | 25.3 (7.43) | 28.5 (6.89) | 29.7 (7.43) | 28.9 (7.87) | 29.8 (7.94) |
|  | Negative Affect | 11.6 (2.50) | 11.4 (1.72) | 10.5 (1.36) | 10.5 (0.90) | 10.3 (0.59) | 10.3 (0.65) |
|  | Hunger | 10.2 (16.8) | 8.04 (9.7) | 5.41 (6.6) | 6.43 (10.6) | 6.10 (6.8) | 6.38 (7.3) |

Values represent mean (*SD*), Hunger rated on Visual Analogue Scales (1-100) and positive and negative affect assessed with Positive and Negative Affect Scales across time points in the total sample (N = 134). BED = Binge Eating Disorder, *n* = 48, OWC = Overweight Control, *n* = 46, NWC = Normal Weight Control, *n* = 40, T = Timepoint.


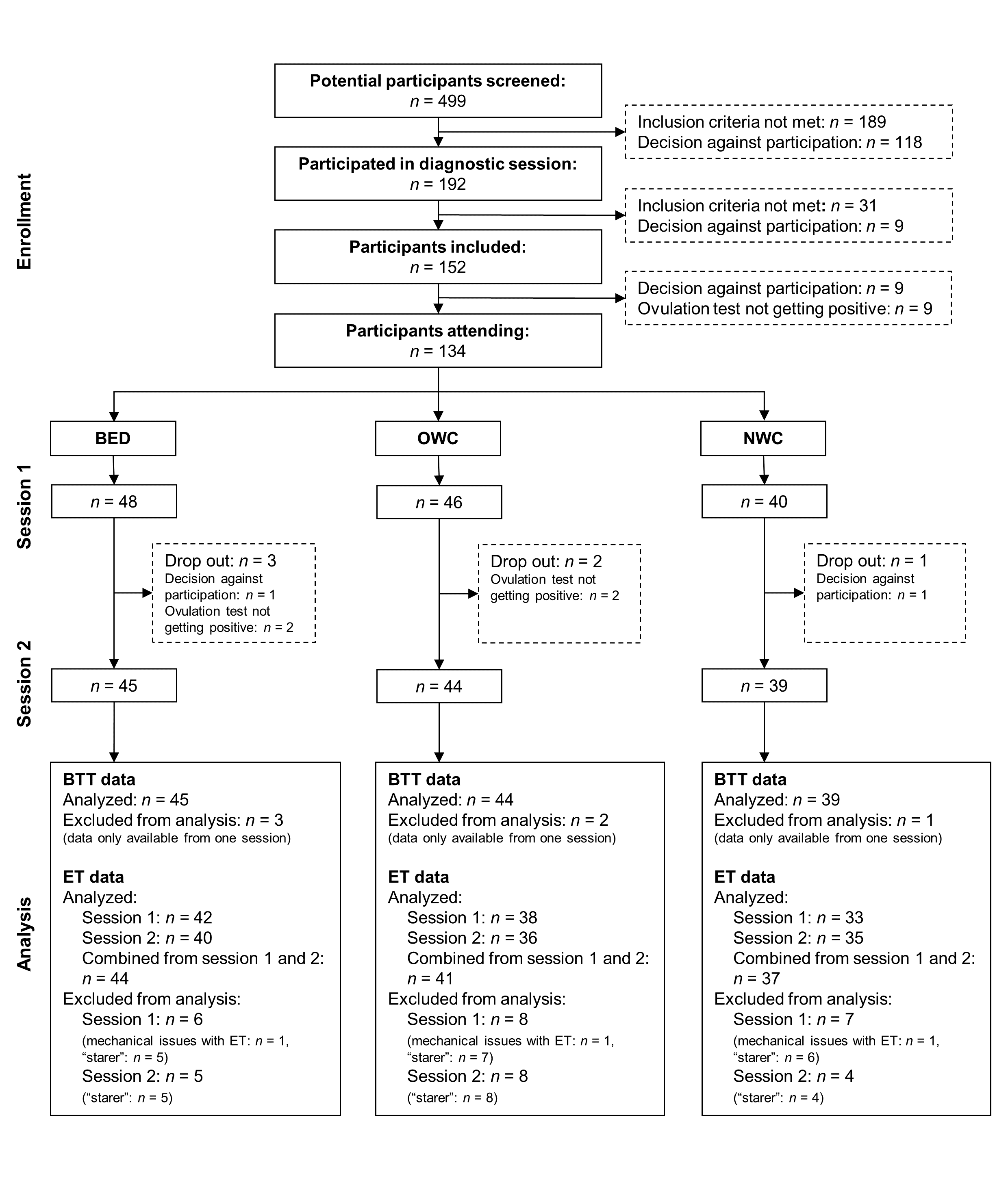


# **Supplementary Figure S1.** Flow of participants. BED = Binge Eating Disorder; OWC = Overweight Control, NWC = Normal Weight Control; BTT = bogus taste test; ET = eye tracking. Vigilance data and dot-probe reaction time data were analyzed for the total sample (*N* = 134).


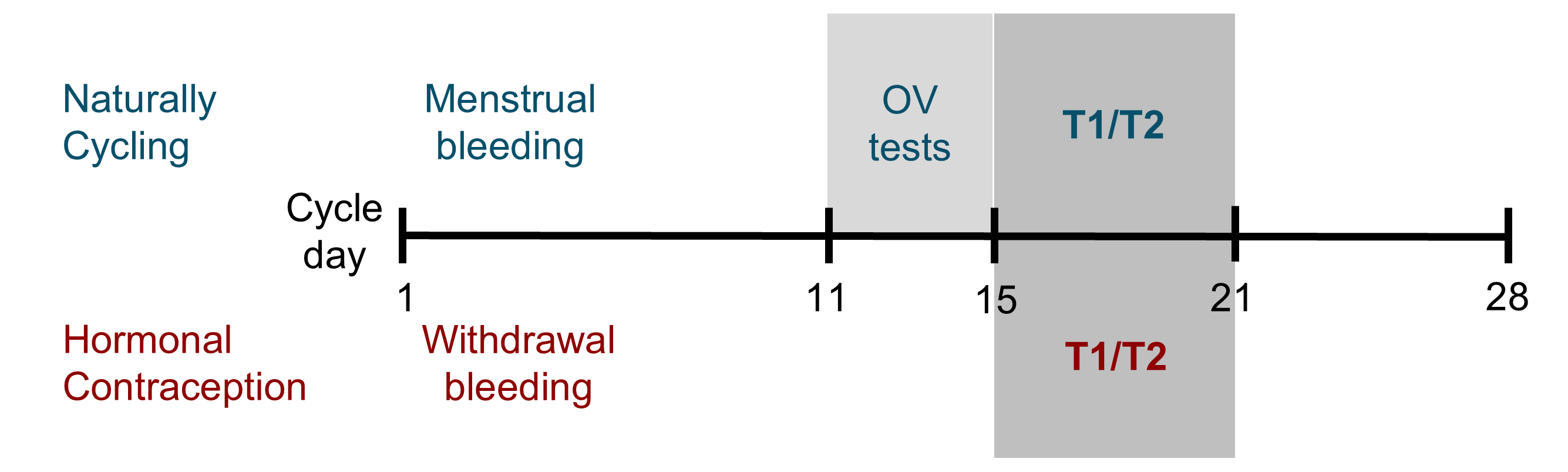


# **Supplementary Figure S2.** Timing of experimental sessions based on hormonal status. Session timing was based on either the natural hormonal cycle or the hormonal contraception cycle for session T1 and session T2 (in the subsequent cycle). In naturally cycling participants, sessions were scheduled two to seven days after a positive ovulation (OV) test result. In post-menopausal participants sessions were spaced apart approximately four weeks. Oxytocin or placebo was administered at either T1 or T2 in a randomized, double-blind design.





# **Supplementary Figure S3.** Reaction times in the vigilance task according to group and condition*.* Depicted are means and standard errors; Total sample, *N* = 134. BED = Binge Eating Disorder, n = 48, OWC = Overweight Control, n = 46, NWC = Normal Weight Control, n = 40. The placebo condition is shown in light gray and the oxytocin condition in red (dark gray).

*

*

# **Supplementary Figure S4.** Reaction times in the vigilance task according to hormonal contraception and condition. Depicted are means and standard errors; Reduced total sample, *n =* 118. No hormonal contraception, *n* = 76, hormonal contraception, *n* = 42. Post-menopausal females (*n* = 16) were not included. The placebo condition is shown in light gray and the oxytocin condition in red (dark gray).

# **Supplementary references**

Bates, D., Mächler, M., Bolker, B. M., & Walker, S. C. (2015). Fitting Linear Mixed-Effects Models using lme4. *Journal of Statistical Software*, *67*(1), 1–48. https://doi.org/10.18637/jss.v067.i01

Castellanos, E., Charboneau, E., Dietrich, M., Park, S., Bradley, B., Mogg, K., & Cowan, R. (2009). Obese adults have visual attention bias for food cue images: evidence for altered reward system function. *International Journal of Obesity*, *33*, 1063–1073. https://doi.org/10.1038/ijo.2009.138

Hautzinger, M., Keller, F., & Kühner, C. (2006). *Das Beck Depressionsinventar II. Deutsche Bearbeitung und Handbuch zum BDI II*. Harcourt Test Services.

Hilbert, A., & Tuschen-Caffier, B. (2016). *Eating Disorder Examination - Questionnaire* (2. Auflage). dgvt-Verlag.

Hilbert, A., Tuschen-Caffier, B., Karqautz, A., Niederhofer, H., & Munsch, S. (2007). Eating Disorder Examination Questionnaire: Evaluation der deutschsprachigen Übersetzung. *Diagnostica*, *53*(3), 144–154.

Khitrov, M. Y., Laxminarayan, S., Thorsley, D., Ramakrishnan, S., Rajaraman, S., Wesensten, N. J., & Reifman, J. (2014). PC-PVT: A platform for psychomotor vigilance task testing, analysis, and prediction. *Behavior Research Methods*, *46*(1), 140–147. https://doi.org/10.3758/S13428-013-0339-9/TABLES/2

Kühner, C., Bürger, C., Keller, F., & Hautzinger, M. (2007). Reliability and validity of the Revised Beck Depression Inventory (BDI-II). Results from German samples. *Nervenarzt*, *78*(6), 651–656. https://doi.org/10.1007/S00115-006-2098-7/TABLES/2

Thompson, B. J., Shugart, C., Dennison, K., & Louder, T. J. (2022). Test-retest reliability of the 5-minute psychomotor vigilance task in working-aged females. *Journal of Neuroscience Methods*, *365*, 109379. https://doi.org/10.1016/J.JNEUMETH.2021.109379

Werle, D., Sablottny, L., Tuschen-Caffier, B., & Svaldi, J. (2024). Modifying biased attention towards food stimuli in binge eating disorder: A multi-session training study. *Appetite*, 107284. https://doi.org/10.1016/J.APPET.2024.107284

Werthmann, J., Jansen, A., & Roefs, A. (2016). Make up your mind about food: A healthy mindset attenuates attention for high-calorie food in restrained eaters. *Appetite*, *105*, 53–59. https://doi.org/10.1016/J.APPET.2016.05.005

Werthmann, J., Simic, M., Konstantellou, A., Mansfield, P., Mercado, D., van Ens, W., & Schmidt, U. (2019). Same, same but different: Attention bias for food cues in adults and adolescents with anorexia nervosa. *International Journal of Eating Disorders*, *52*(6), 681–690. https://doi.org/10.1002/eat.23064
